# Concern for *Dirofilaria immitis* and Macrocyclic Lactone Loss of Efficacy: Current Situation in the USA and Europe, and Future Scenarios

**DOI:** 10.3390/pathogens10101323

**Published:** 2021-10-14

**Authors:** Anastasia Diakou, Roger K. Prichard

**Affiliations:** 1 School of Veterinary Medicine, Faculty of Health Sciences, Aristotle University of Thessaloniki, 54124 Thessaloniki, Greece; 2 Institute of Parasitology, Faculty of Agricultural and Environmental Sciences, McGill University, Sainte Anne-de-Bellevue, QC H9X3V9, Canada

**Keywords:** *Dirofilaria immitis*, macrocyclic lactones, resistance, diagnosis, treatment, prevention

## Abstract

*Dirofilaria immitis* infection is one of the most severe parasitic diseases in dogs. Prevention is achieved by the administration of drugs containing macrocyclic lactones (MLs). These products are very safe and highly effective, targeting the third and fourth larval stages (L3, L4) of the parasite. Until 2011, claims of the ineffectiveness of MLs, reported as “loss of efficacy” (LOE), were generally attributed to owners’ non-compliance, or other reasons associated with inadequate preventative coverage. There was solid argumentation that a resistance problem is not likely to occur because of (i) the great extent of refugia, (ii) the complexity of resistance development to MLs, and (iii) the possible large number of genes involved in resistance selection. Nevertheless, today, it is unequivocally proven that ML-resistant *D. immitis* strains exist, at least in the Lower Mississippi region, USA. Accordingly, tools have been developed to evaluate and confirm the susceptibility status of *D. immitis* strains. A simple, in-clinic, microfilariae suppression test, 14-28 days after ML administration, and a “decision tree” (algorithm), including compliance and preventatives’ purchase history, and testing gaps, may be applied for assessing any resistant nature of the parasite. On the molecular level, specific SNPs may be used as markers of ML resistance, offering a basis for the validation of clinically suspected resistant strains. In Europe, no LOE/resistance claims have been reported so far, and the existing conditions (stray dogs, rich wildlife, majority of owned dogs not on preventive ML treatment) do not favor selection pressure on the parasites. Considering the genetic basis of resistance and the epizootiological characteristics of *D. immitis*, ML resistance neither establishes easily nor spreads quickly, a fact confirmed by the current known dispersion of the problem, which is limited. Nevertheless, ML resistance may propagate from an initial geographical point, via animal and vector mobility, to other regions, while it can also emerge as an independent evolutionary process in a new area. For these reasons, and considering the current chemoprophylaxis recommendations and increasing use of ML endectoparasiticides as a potential selection pressure, it is important to remain vigilant for the timely detection of any ML LOE/resistance, in all continents where *D. immitis* is enzootic.

## 1. Introduction

*Dirofilaria immitis* (Filarioidea: Onchocercidae) is a nematode parasite that inhabits the pulmonary arteries of dogs and other carnivores, including cats. Under specific circumstances, such as a rise in pulmonary artery pressure, entanglement in chordae tendineae, heavy parasitism, and nullification of pulmonary artery pressure after host death, these parasites can be also found in the right chambers (ventricle and atrium) of the heart and for this reason are commonly known as “heartworms”. *Dirofilaria immitis* is the agent of dirofilariosis (heartworm disease), one of the most significant, potentially fatal parasitic diseases in dogs. It has a worldwide distribution, with higher prevalence in temperate and subtropical zones, and it shows a trend of expansion in cooler climates and in areas that were previously considered heartworm-free [1,2]. As a key example, in Europe, infections are now expanding northward from the previously known enzootic areas [3], while, at the same time, the parasite is establishing in southern areas that were considered free or reported only sporadic cases in the past [4].

*Dirofilaria immitis* has an indirect lifecycle and is transmitted by the bite of infected mosquitoes. Over 60 species of mosquitoes have been identified as potential intermediate hosts/vectors of *D. immitis* [5]. Mosquitoes, in the course of a blood meal from a definitive host, ingest the microfilariae, i.e., the stage produced by adult female heartworms, that circulate in the bloodstream of infected animals. Microfilariae develop inside the arthropod to first-stage larvae (L1) and then molt twice, to second (L2), and finally to the infective, third-stage larvae (L3), in a period of 8–29 days, depending on the environmental temperature and mosquito species [2,6]. The infective larvae migrate to the proboscis of the mosquito and can be transmitted during another blood meal to a mammalian host, as they pass in a pool of mosquito hemolymph, deposited at the site of the bite, and enter the definitive host through the wound [2,5]. Inside the definitive host, L3 remain close to the site of inoculation and molt to the fourth-stage larvae (L4) in 4–23 days post-infection (dpi). The latter stage migrates in the subcutaneous and intramuscular tissues and finally molts to the juvenile adult stage between 50 and 58 dpi. By day 70 pi, the first parasites arrive in the pulmonary artery and by day 120 pi, most parasites have reached their final site of parasitism. The first microfilariae appear in the circulation of the infected animal around 180 dpi [2]. 

Heartworm infection in dogs may remain subclinical but often leads to clinical disease, associated mainly with pulmonary hypertension caused by the structural alterations of the arteries in the presence of worms. Mechanical and immunological factors result in thickening, hardening, and narrowing the pulmonary arteries, which become inelastic, leading to core pulmonale and eventually heart failure. The pathogenic action of *D. immitis* depends on factors such as the number of worms present in the pulmonary arteries, the size of the dog in relation to this number, the individual immune response to the infection, and the level of activity of the dog. Overall, heartworm disease may manifest with exercise intolerance, cough, hemoptysis, respiratory distress (tachypnea, dyspnea), syncope, and, in severe cases, with pulmonary thromboembolism, caval syndrome (where the worms are trans-placed back to the right chambers of the heart and the vena cava, causing valvular disfunction, blood flow impairment, hemolysis, liver, kidney, and heart failure), and death [7].

The infection in cats displays some particularities, as cats are not considered a natural host for *D. immitis*. The parasites are usually very few in feline hosts and they often do not develop into reproducing mature worms [8]. Although, in enzootic areas, cats are at risk of infection, it is estimated that only around 5–20% of the prevalence found in dogs of the same area corresponds to cat infection [9], although, under specific conditions of close and restricted symbiosis with dogs (e.g., in an animal shelter), this analogy may be higher [10]. The lifespan of heartworms in cats is shorter than in dogs (2–3 years vs. 5–7 years) and the infection usually remains asymptomatic but may result in sudden death after the death of the parasites, because of thromboembolism but mostly because of an anaphylactic immune reaction. Cats may also develop a severe and often fatal lung pathology (vascular and parenchymal inflammatory response), associated with the death of newly arriving immature worms, known as “heartworm associated respiratory disease” (HARD) [2,8]. 

Heartworm treatment is complicated, expensive, and encompasses risks as the dying parasites can cause thromboembolism that may result in severe implications. For this reason, a strict restriction of activity is recommended for the dogs, from the day of diagnosis until the end of treatment. In some cases, stabilization of the dog’s health condition prior to treatment is necessary, while, in other cases, surgical removal of the worms is the best option. The treatment protocol as proposed by the American Heartworm Society (AHS) takes at least 5 months to be completed, including exercise restriction after the last adulticide drug administration [11]. On the other hand, there is no approved treatment protocol for feline dirofilariosis. In cases where there are no clinical signs, waiting for the natural, spontaneous death of the parasites may be the best choice, while supportive treatment with corticosteroids may be considered necessary in the case of symptomatic infection [12]. 

*Dirofilaria immitis* has zoonotic potential and humans living in enzootic areas are at risk of infection. The parasite does not mature in humans, but, usually, after reaching the pulmonary artery, dies, causing pneumonitis and, subsequently, the formation of a spherical granuloma (“coin lesion”). Human pulmonary dirofilariosis may remain asymptomatic or manifest clinically with cough, chest pain, blood-tinged sputum, low-grade fever, and eosinophilia [13]. The impact of pulmonary dirofilariosis on human health is not associated so much with the pathology caused by the parasite but rather with the differential diagnosis of the associated lesions. In fact, a “coin lesion” will trigger an investigation for neoplasias (primary or metastatic), hamartomas, tuberculosis, and fungal infections. The etiological diagnosis requires a long, laborious clinical and laboratory work-up, and usually is only achieved after surgery and histopathological examination of the exerted granuloma. These procedures are of high economical and, most importantly, psychological and health costs to the patient, especially in the case of unnecessary, invasive procedures, which may have severe side implications [13]. 

Due to the impact of heartworms on the health of pets, the complexity, risk, and cost of the treatment, and the zoonotic implications, heartworm prevention in dogs is imperative. Currently, there are many veterinary products in the market approved for this purpose and all of them contain a single drug class, i.e., the macrocyclic lactones (MLs) (Table 1). These drugs, when administered according to label instructions, are very safe and effective at preventing heartworm disease. In fact, there is evidence that, in highly enzootic areas, where prevention has been applied systematically, the occurrence of infection among dogs that are not under preventive drugs drops significantly [14,15]. The efficacy percentage in the experimental trials for the approval of heartworm-preventive drugs is set at 100% [16], and even though MLs reach this level of efficacy in registration studies, some *D. immitis* strains have been unequivocally proven resistant to these molecules. The problem of possible resistance to MLs emerged in the early 2000s and was confirmed approximately 10 years later. To date, the problem is restricted to a specific area of the USA and it is monitored by ongoing surveys and case investigations. The present article aims at providing an overall view of *D. immitis* resistance development to ML preventives, as a challenge to the veterinary, academic, and industry world. The following sections deal with (i) the past and present state of heartworm prevention, (ii) the target of MLs as preventives, (iii) the existent knowledge of MLs’ mode of action and related resistance development by parasites, (iv) the history of loss of efficacy (LOE) reports, (v) the chronicle of *D. immitis* resistance confirmation, (vi) the tools developed for resistance detection in clinical and laboratory settings, (vii) the current situation in the USA and Europe, (iix) possible scenarios about the evolution of the problem, and, finally, (ix) some practical suggestions and strategies for the monitoring, detection, and prevention of the problem.

## 2. Past and Present State of Heartworm Prevention

The first preventive for canine heartworm was the piperazine derivative diethylcarbamazine (DEC), which entered the market in 1977. In order to be effective, DEC had to be administered daily, as it affects the L3-L4 molt that occurs a few days post-infection [17]. Apart from the practical inconvenience of daily administration, DEC displayed serious systemic adverse effects when administered to microfilaremic dogs, due to anaphylactic reaction, while it is also partially macrofilaricidal and may induce adverse effects caused by the death of adults [18]. 

The first evidence that MLs display activity against *D. immitis* came with the investigations of Campbell and Blair in 1978 [19]. The launch in 1987 of the first monthly administered veterinary product containing ivermectin, being very safe and effective against heartworm infection, represented a powerful alternative to DEC. In the following years, a variety of veterinary products, including topical and parenteral forms, offered a number of choices and dominated the market [20].

Currently, chemoprophylaxis against heartworm disease is achieved with the administration of drugs belonging to the ML class. The molecules in products licensed for this purpose are ivermectin (IVM), selamectin (SEL), eprinomectin (EPR), and abamectin (ABA) (licensed in Australia for use in dogs) from the group of avermectins and milbemycin oxime (MO) and moxidectin (MOX) from the group of milbemycins. There are many forms available on the market, i.e., spot-ons, chewables, tablets, and injectables, some of them as single active ingredient products but most of them as combinations of antiparasitics, often in the form of endo-ectoparasiticides. The majority of these formulations are licensed for heartworm prevention by monthly administration but there are also injectable forms of MOX for administration every 6 or every 12 months (extended-release injectable forms) (Table 1). 

**Table 1:** Veterinary products with macrocyclic lactones, registered in the USA or Europe for heartworm prevention in dogs and cats *.

Active Molecule **	Target Species	Application Route/Administration	Product/Company	Combination Molecule(s)
Eprinomectin	cat	topical/monthly	Centragard ^2^/Boehringer lngelheim	Praziquantel
			NexGard Combo ^3^/Boehringer lngelheim	Esafoxolaner, Praziquantel
			Broadline ^3^/Boehringer lngelheim	Fipronil, Praziquantel, (S)-Methoprene
Ivermectin	dog, cat	oral/monthly	Heartgard ^2^/Boehringer lngelheim Iverhart ^2^/Virbac Ivermectin ^2^/Cronus Pharma	-
	dog	topical/monthly	Advantage DUO ^2^/Elanco	Imidacloprid
		oral/monthly	Heartgard Plus ^2^/Boehringer lngelheim Iverhart Plus ^2^/Virbac Tri-Heart Plus ^2^/Heska	Pyrantel
			Panacur Plus ^2^/Intervet	Praziquantel, Fenbendazole
			Iverhart Max ^2^/Virbac	Praziquantel, Pyrantel
			Heartgard Plus ^3^/Boehringer lngelheim	Pyrantel
			Cardotek Plus ^3^/Boehringer lngelheim	
			Cardotek ^3^/Boehringer lngelheim	-
Milbemycin oxime	dog, cat	oral/monthly	Interceptor ^1^/Elanco MilbeGuard ^2^/Ceva Sante Animale	-
			Interceptor Plus ^1^/Elanco Milbemax ^3^/Elanco Milbactor ^3^/Ceva Sante Animal Milprazon ^3^/Krka Milquantel ^3^/Krka Milpro ^3^/Virbac	Praziquantel
	dog	oral/monthly	Sentinel ^2^/Intervet Program plus ^3^/Elanco	Lufenuron
			Sentinel Spectrum ^2^/Intervet	Lufenuron, Praziquantel
			Interceptor Plus ^2^/Elanco	Praziquantel
			Trifexis ^1^/Elanco	Spinosad
			NexGard Spectra ^3^/Boehringer lngelheim	Afoxolaner
			Credelio Plus ^3^/Elanco	Lotilaner
Moxidectin	dog, cat	topical/monthly	Prinovox ^3^/Virbac	Imidacloprid
			Advantage Multi ^2^/Elanco	
			Imoxi ^2^/Vetoquinol	
			Advocate ^3^/Elanco	
	dog	oral/monthly	Simparica Trio ^1^/Zoetis	Sarolaner, Pyrantel
			ProHeart ^2,4^/Zoetis	-
		inj./6 month	Proheart 6 ^2^/Zoetis	
			Guardian ^3^***/Elanco	
			Afilaria ^3^/Fatro, Support Pharma	
		inj./12 month	Proheart 12 ^2^/Zoetis	
		topical/monthly	Coraxis ^2^/Elanco	
	cat	topical/monthly	Bravecto Plus ^1^/Intervet	Fluralaner
Selamectin	dog, cat	topical/monthly	Revolution ^2^/Zoetis	-
			Revolt ^2^/Aurora Selarid ^2^/Norbrook Lab. Senergy ^2^/Chanelle Stronghold ^3^/Zoetis Chanhold ^3^/Chanelle Evicto ^3^/Virbac Stronghold Plus ^3^/Zoetis	Sarolaner
	cat	topical/monthly	Revolution Plus ^2^/Zoetis Stronghold Plus ^3^/Zoetis Felisecto Plus ^3^/Zoetis	

* Information retrieved from the European Medicines Agency (https://www.ema.europa.eu/en, accessed the 5th of August 2021), the U.S. Food and Drug Administration (https://animaldrugsatfda.fda.gov/adafda/views/#/search accessed the 5th of August 2021), and from [5] for Europe and the USA. ** For heartworm prevention. *** To be administered yearly, the first month of mosquito activity, according to the drug instructions in Europe. ^1^ registered in USA and Europe. ^2^ registered in USA only. ^3^ registered in Europe only. ^4^ registered in the USA, but no longer available

## 3. Effect of MLs on *D. immitis*

It has been shown that MLs are highly effective against the L3 and L4 stages of *D. immitis* and kill them rapidly, at low concentrations. For example, IVM at the dose rate of 6 μg/kg per os can clear these early stages from the day they enter the host up to 60 days pi [21]. During this period, the parasites have reached the L4 stage but are still migrating in the connective tissue and most of them have not yet entered the blood vessels [22]. However, MLs do not display prospective or residual efficacy against *D. immitis* and, simply put, they have no “forward” action (against future infections) but rather a “reach-back” efficacy (against past inoculations). Thus, the strategy of the periodic administration is based on the realistic scenario that dogs are under continuous exposure to infective mosquito bites throughout the period of transmission and that monthly administration of MLs ensures that no worms will live to reach the pulmonary arteries, even in the case of dosing delayed by a few days [23].

Furthermore, other than preventing the establishment of infection by killing L3 and L4, MLs have also an effect on young adult and adult worms, but this action is apparent after several, continuous, periodic administrations of the drugs. More precisely, in the case of owner compliance failure or of missed/low dosing for any other reason, monthly administration of prophylactic doses of IVM gradually eliminates all parasite stages and effectively prevents or greatly reduces the possibility of heartworm disease establishment [24], a process known as “slow-kill”. As IVM was the first licensed ML for heartworm prevention, it is the most studied molecule, and although the rest of the MLs have been relatively less explored, the available data show that not all MLs are equally effective against worms older than 30 days. For example, the injectable sustained-release formulation MOX, licensed for administration every 6 months, was highly effective after a second injection at 6 months; SEL was also highly effective against 3-month-old heartworms when given monthly for 1 year in prophylactic doses, while MBO was less effective against 4-month-old worms after a year of monthly administration [24]. These data are the basis of the observed “safety net”, i.e., the fact that the continuous, periodic administration of MLs in preventive doses can safely protect dogs from developing heartworm disease. Accordingly, the sooner after the infection the consistent ML administration starts, i.e., the younger the parasites are, the more complete and fast the elimination of the parasites. Finally, there is also an effect of MLs on microfilariae and this again varies between the different molecules, dose rates, and formulations. IVM at a high dose rate and MOX, perform best at eliminating circulating microfilariae. However, only MOX is licensed as a microfilaricidal drug [25].

## 4. MLs’ Mode of Action and Resistance to MLs

The pharmacological mode of action of MLs on different stages of *D. immitis* is not conclusively decoded. However, there is some knowledge on MLs’ mode of action gathered from other nematode parasites. For example, based on the genetic changes found in *Haemonchus contortus* and *Cooperia oncophora* with a resistant phenotype to MLs, there is evidence that these molecules act on glutamate-gated chloride (GluCls) and γ-aminobutyric acid (GABA) chloride channels, P-glycoprotein (Pgp), ABC transporters, and β-tubulin [26]. These are receptors present in many cells of the nematodes, regulating locomotion and reproduction [27]. When MLs bind to these receptors, these channels open, causing hyperpolarization of the parasite’s cells, which leads to flaccid paralysis, which is lethal for parasites such as gastrointestinal nematodes, which, in such a state, can be expelled from the host [28]. Furthermore, in nematodes that take nutrients via the mouth opening, paralysis of the pharyngeal muscle cells leads to their starvation and death [29]. 

It is known that filarial nematodes have the abovementioned ligand-gated chloride channels [30] and there is evidence that MLs cause paralysis to microfilariae in vitro [31]. However, filarial nematodes have critical anatomical and physiological differences from other nematodes. For example, filarial worms absorb nutrients through the cuticle while their pharynx is vestigial. Furthermore, the sites where filarial nematodes parasitize would permit them a period of muscle paralysis without being physically removed from the host. On the other hand, their reproduction is more prominently affected by MLs than it is in non-filarial nematodes [27]. Consequently, in order to understand MLs’ mode of action on *D. immitis*, it is more relevant to consider any known action of these molecules on parasites of close genetic relation, i.e., other nematodes of the family Onchocercidae. 

Indeed, there is evidence that MLs disrupt the function of the excretory–secretory organ in larval stages and microfilariae of *Brugia malayi* (agent of lymphatic filariosis) by paralyzing the excretory pore cells. This leads to less effective secretion of immunomodulatory substances by the parasites, which then become vulnerable to the immunological mechanisms of the host [32]. Furthermore, MLs are capable of suppressing reproduction in *B. malayi* by interfering with both the female and male reproductive system. This has been suggested because CluCl channel signals were detected in important elements of these systems, i.e., the ovary, embryos, lateral hypodermal chords, uterus wall, spermatogonia, vas deferens wall, and somatic muscles adjacent to the terminal end of the vas deferens [33]. By affecting the muscle cells of all these sites, MLs (a) suppress microfilariae production and (b) may result in adult worm death after repeated doses [34].

The development of resistance against MLs has been documented in various nematodes. The resistance of parasites to drugs is a genetic characteristic (and thus heritable); therefore, a selection in a parasite population for anthelmintic resistance would be reflected in a selection for particular genes that encode this characteristic [27]. According to Prichard et al. [35], resistance has occurred in a population when there is a greater frequency of parasites—compared to the frequency in a normal population of the same species—able to survive in the presence of a specific drug dose. 

Filarid nematodes of medical importance have been investigated for resistance development. Although, in these nematodes, resistance is not as common as, for instance, in gastrointestinal nematodes of farm animals, it has been shown that in *Onchocerca volvulus* and *Wuchereria bancrofti*, agents of river blindness disease and lymphatic filariosis, respectively, genetic changes are consistent with selection pressure [36]. For example, in *O. volvulus*, resistance is not related to selection on GluCl or GABA genes, but selection on tubulin, Pgp, and other ABC transporter genes has been found (reviewed in [37]). Whether the knowledge acquired from the volume of work accomplished on other filarid nematodes is applicable to *D. immitis* genetic adaptation in the face of selection pressure is a question yet to be answered. 

## 5. First Reports of Loss of Efficacy (LOE) in the USA: Interpretations and Skepticism

Based on the records of the Center for Veterinary Medicine, U.S. Food and Drug Administration (FDA/CVM), the first complaints of ML LOE came to light in 1998, a decade after their introduction to the market as heartworm preventives [20]. However, these early reports were not well documented and the vast majority were attributed to a lack of owner compliance. Nevertheless, in the following years, the number of reported LOE cases rose, with peak of numbers in 2002. This event, in combination with the rise of mosquito concentrations and abundance due to the Gulf Coast weather changes (numerous tropical storms and the extreme phenomenon of the Katrina hurricane) in the period of 2001-2005, led to regulations of closer monitoring of LOE cases by the FDA/CVM, and finally to a report of a possibly newly emerging resistance problem by Victoria Hampshire at the FDA/CVM [20]. In this article, a scoring system from 0, representing a remotely possible true LOE, to 6, representing a very possible true LOE, was proposed in order to evaluate the reliability of each case report. This scoring was based on three main pillars: (a) the mode of HW prevention application, i.e., seasonal or year-round, (b) if testing was performed before initiation of preventives and the timing of the subsequent tests, and (c) the age of the dogs when prevention started, in combination with the season of birth for dogs under 1 year of age [20].

As claimed in this first analysis about possible LOE (resistance emergence) [20], the factor of owner compliance was rather decisive for a great number of the LOE cases reported. Indeed, Atkins et al. [38] investigated this issue in a retrospective medical record review. The investigation included records from veterinary practices in the area of the Mississippi Delta, as this was the area where most of the LOE complaints originated. According to the results, only 5 of the 301 suspected cases, assessed in the survey, had no factors disrupting prevention, while most LOEs were associated with likely failure to meet heartworm prevention recommendations. This category of infections included the cases of owner (or possibly veterinarian) non-compliance, i.e., missed or late doses, doses that had been shared among pets of the same household, a lack of testing before the first preventive treatment, and inadequate follow-up tests, and also cases of insufficient drug concentration in the dog because of an incidence of vomiting or excessive diarrhea (for the per os administered products). In any case, they did not represent a genuine resistance problem [38].

It is also possible that a policy of the pharmaceutical companies, known as “customer satisfaction programs” or “guarantees”, may have also played a role in falsely raising the number of LOE reports. According to this policy, the companies provided support for the treatment of dogs that became infected and for which their preventive product was given to the pet owner. The criteria for providing this assistance were generally loose and it was mainly required that a dog received the company’s heartworm-preventive product during the previous year and was heartworm antigen-negative before that. Although these criteria are not enough to indicate that the product actually failed in protecting the animal, all the cases that fell into the customer satisfaction program were, obligatorily, reported to the FDA/CVM. This raised the number of LOE cases in the authorities’ records [38].

Based on the abovementioned analyses and interpretations, and considering the factors reported by Prichard [27] that may play a decisive role in parasite drug resistance (see Section 10), the emergence of resistance in *D. immitis* had, up to a certain time point, been considered unlikely [39].

## 6. Confirmation of *D. immitis*-Resistant Strains

After the first reports of suspected ML LOE [20], and despite the evidence that most of these cases were actually due to insufficient preventive coverage of the dogs [38], the first unequivocally resistant strains of *D. immitis*, originating from the Lower Mississippi area, were genetically, in vitro, and clinically confirmed [37,40]. Indeed, by comparing parasites from laboratory lineages with known susceptibility to MLs, evidence was generated at the molecular level. It was shown that parasites implicated in LOE cases were characterized by a very high occurrence of specific single-nucleotide polymorphisms (SNPs) and a loss of heterozygosity in a gene encoding a P-glycoprotein transporter, with homozygous guanosine residues at two locations, which became known as the “GG-GG” genotype [37]. The high frequency of homozygosity in these parasites could be attributed to the non-random mating in the examined *D. immitis* population, a phenomenon observed in drug selection, where the resistant parasites dominate in the population. The microfilariae of these GG-GG genotype strains also showed very low in vitro sensitivity (lethality) in the presence of IVM, compared to a known laboratory-susceptible strain, phenotypically confirming their resistant nature. Interestingly, the percent mortality was inversely proportional to the GG-GG percentage of the strain [37]. This diagnostic approach was applied to an additional suspected clinical case and was further validated [41].

Soon, the in vivo, clinical confirmation of ML-resistant *D. immitis* strains followed. Pulaski et al. [40] successfully infected laboratory dogs treated with the monthly administration of IVM for 6 months at twice the label dose, with parasite strains isolated from field cases originating from Louisiana and fitting the criteria for LOE suspicion. Further, in order to prove the heritability of the characteristic, a second resistant parasite generation was developed in dogs on prophylactic ML administration [40]. 

Although it was clear that further investigations on the parasite’s genome and the discovery of additional, reliable genetic markers of resistance was warranted, these pioneer surveys unequivocally demonstrated the existence of a resistance problem in *D. immitis* populations.

## 7. Tools Developed for Resistance Detection

After unambiguously confirming the existence and establishment of *D. immitis* resistance to MLs, the next step would be to develop clinical and laboratory tools that could serve in detecting and confirming new cases of infection by resistant strains. Such tools should be simple, reproducible, and inexpensive, to allow monitoring of the prevalence and distribution of resistant strains [42]. In this direction, several attempts have been made, of which some resulted in useful tools and protocols while others were not equally successful in proposing reliable and practical tests and methods. The procedure for detecting an infection by a resistant strain usually starts in the clinic and, at least to date, can only be completed by confirmation in the laboratory. The attempts and achievements on detecting *D. immitis* resistance to MLs are highlighted in the following subsections.

### 7.1. In the Clinic

Although, currently, advanced laboratory tests are necessary to prove the resistant nature of a strain, they are laborious and expensive and, as such, they cannot be widely applied in all suspected cases. Indeed, L3, i.e., the parasite stage on which MLs are primarily effective and act as preventives, are not easily available in large numbers for laboratory trials of drug effectiveness [36]. Similarly, access to suspected drug-resistant parasites derived from cases diagnosed in veterinary practices is also limited due to restrictions related to legislations and, of course, ethical and emotional implications. Furthermore, the experimental, in vivo confirmation of the resistance status of the parasites (i.e., the experimental infection of laboratory dogs under preventives; see Section 6, [40]) is time-consuming, costly, and ethically questionable [36]. Thus, there was a clear need for the development of a simple trial that could be performed in-clinic and that would provide reasonable evidence towards the susceptibility status of the parasites involved in any resistance-suspected case of heartworm infection. 

An in vivo trial fulfilling this need was proposed by Geary et al. [36] and it has become known as the Microfilariae Suppression Test (MFST). It is based on the observation that MLs have an effect on microfilariae and reduce their number or even totally eliminate them, even in cases of fertile adult heartworms existing in the pulmonary arteries [36]. In short, in every microfilaremic dog suspected of infection by resistant parasites, i.e., which, according to its medical records and history, became infected despite consistent chemoprophylaxis, the Knott’s test is performed, and microfilariae are counted per mL of blood. Immediately after, a microfilaricidal dose of an ML is administered. In Geary et al. [36], IVM, at the dose of 50 or 200 μg/kg depending on the dog breed, is proposed for this purpose, while MOX at the dose of 2.5 mg/kg in a product licensed as microfilaricidal (Advantage^®^ Multi, Advocate^®^) has been also used in this context [43]. The microfilarial count was suggested to be repeated by the Knott’s test 7 days later [36] or, as applied in Ballesteros et al. [43], 2–4 weeks after dosing, in accordance with the findings of Bowman et al. [25] that the microfilaremia is only significantly reduced after this interval. As MOX is the only product with registered microfilaricidal efficacy, the use of the registered product is recommended for this test. Furthermore, based on the effects of MOX on microfilariae [25], the post-treatment blood microfilarial count is recommended to be 2–4 weeks after treatment and not 1 week, as false positives for putative resistance are likely to be high if the post-treatment count is too early. In the event that, after this time lapse, the microfilariae counts show a reduction of ≤75%, a resistant strain is probable. Although it cannot be ruled out that, in some cases, a reduction of ≤75% may not be an accurate indication of a factually resistant strain [44], it is important to stress that a reduction in microfilariae number close to 100% indicates ML-susceptible parasites [36,43].

### 7.2. In the Laboratory

Laboratory trials based on the phenotype and biological characteristics of the isolated strains have been developed in an attempt to assess the resistant character. These include in vitro bioassays that assess the mortality/mobility and biochemical phenotype of accessible parasite stages. It is common that, for the in vitro assays, surrogate stages of the parasites, rather than the actual drug target stages, are used, because they are often easier to obtain and manipulate in the laboratory [42]. In the case of *D. immitis*, the most accessible and easy life stage to work with are the microfilariae, although the infective stage L3 have been also implemented in laboratory trials [39,45]. However, important drawbacks have been observed in assays implementing the in vitro assessment of the efficacy of MLs. For example, ML concentrations >6000 times higher than those reached in vivo did not achieve 100% motility inhibition, i.e., inhibition of migration through a mesh, in a susceptible *D. immitis* strain. This observation led to the assumption that the effectiveness of MLs’ action in vivo is not on parasite motility but involves the immune response of the host [39]. Furthermore, when comparing susceptible with resistant parasites by the L3 migration inhibition assay, no statistically significant differences in drug susceptibility were observed, suggesting that ML resistance in *D. immitis* is not related to the paralysis of L3, rendering such assays inappropriate for detecting resistant strains [45].

Similarly, a comparative in vitro microfilariae motility assessment in susceptible and resistant strains showed that motility measurements are not reliable for discriminating ML resistance and that MLs probably do not kill microfilariae by paralyzing them [42]. 

The biochemical phenotype of *D. immitis* strains has also been a subject of analysis as a potential indicator of resistance against MLs. The cell membrane integrity and the metabolic activity were assessed with the use of vital stains (trypan blue, propidium iodide staining, and resazurin), and the P-glycoprotein-mediated efflux was studied in susceptible and ML-resistant strains. The results showed that there is a natural variability in these traits between different *D. immitis* isolates and that none of these methods can be used to accurately detect the factual drug susceptibility status [46].

### 7.3. Molecular Tools

At the molecular level, the existence of resistant strains could be investigated in different ways. As reported by Geary et al. [36], one way would be the detection of lower heterozygosity in the suspected population of parasites, compared with a population of the optimal (susceptible) phenotype. However, if an infection in only a single dog is being investigated, there may be little heterozygosity as the population size of the single infection may be low. Therefore, this is not a practical approach. Another attempt would be the detection of specific SNPs that appear in higher prevalence in known resistant populations. Finally, a whole-genome analysis, comparing known susceptible and phenotypically resistant populations, would provide relevant information. 

Based on these options, a significant volume of research has been conducted (reviewed in [36]). These investigations, based on the whole-genome analysis of *D. immitis* [47], led to the identification of genetic markers apparently associated with ML resistance. Initially, 186 SNP loci across the whole *D. immitis* genome, that could indicate ML-resistant strains were identified, then reduced to 42 [48], and later narrowed down to three SNPs that appeared suitable for future monitoring [49]. Most recently, in an attempt to identify specific SNP patterns associated with ML resistance, which would reflect the phenotype of MF response to a microfilaricidal ML, i.e., a relatively simple challenge test that could be applied in clinical cases, 10 SNPs, previously identified to better differentiate the ML-susceptible phenotype from the resistant ones [49], were used and applied in the analysis of microfilariae from 50 different cases with different phenotype responses to MLs [43]. According to the results, there was a significant genotype–phenotype association between SNPs detected in ML-resistant strains, i.e., those that did not respond with microfilaremia reduction after ML administration. The results of Ballesteros et al. [43] suggest that ML resistance may be a polygenic trait and, importantly, that there is probably a spectrum of resistant phenotypes. In this study, a specific two-SNP model was found to be equally effective as the 10-SNP model in the samples examined. The molecular analysis of these selected SNPs is currently the best available diagnostic tool for the confirmation of clinically suspected cases.

## 8. Current Situation in the USA

According to the most recent information, resistant strains have been identified so far only in the Lower Mississippi region [37,40,41,43]. Indeed, the first genetically confirmed resistant strain originated from Arkansas and Louisiana [37]. The GG-GG genotype was then detected in a dog infected in the area of New Orleans [41]. Further resistant cases were detected in the Mississippi Delta by Pulaski et al. [40,48] and Lower Mississippi region by Ballesteros et al. [43]. 

The reason that resistance against MLs in *D. immitis* has developed in this particular area of the world is not totally clear. Speculation may be made about possible favorable conditions that occurred in the specific area or/and in the specific timeframe when the first cases appeared. Local conditions, i.e., the climate, wetlands, and mosquito abundance in the area, favor *D. immitis* transmission and there is evidence that, when parasite transmission is high, resistance development is facilitated [50]. Furthermore, extreme natural rainfall phenomena in the area—and, in particular, Hurricane Katrina, with the devastation and massive mosquito bloom—likely played a critical role in enhancing conditions, favoring infection pressure. It could be suggested that, under such conditions and in combination with intensive treatments with ML heartworm preventives, resistant strains of *D. immitis* are more likely to spread. 

## 9. Current Situation in Europe

To date, there are not any confirmed resistant strains circulating in Europe. However, there has been a small number of cases that generated strong suspicion of resistance presence [44]. These suspected cases were detected in Greece, considering seven dogs with detailed records of consistent monthly preventive administration and only a single missed dose, recorded 2 years earlier in three of them. In three of these infected dogs, the MFST was applied with the administration of IVM (200 μg/kg). The MFST was repeated for the subsequent 9 months of the total 10 months of monitoring, where microfilariae were counted every month immediately before and 7 days after IVM administration. Microfilariae showed a relatively stable count until the 8th month of monitoring. In most cases, the count reduction was <75% after each IVM administration and, interestingly, in some cases, the counts even escalated. However, this indication of possible resistant *D. immitis* isolates was not genetically confirmed: microfilariae from all seven dogs from Greece, as well as adult *D. immitis* worms, after the sudden death of one of them, were isolated and subjected to MiSeq next-generation sequencing of regions encompassing the 10 SNPs previously identified as highly correlated with ML resistance [43]. The variance of the allele frequency at a given SNP position was compared to previously described allele frequencies for resistant and susceptible populations and revealed that the examined isolates were highly consistent with confirmed ML-susceptible samples. In contrast, the one known resistant USA isolate analyzed in parallel showed a genotype confirming ML resistance [44].

Recently, an investigation of eleven European *D. immitis* clinical isolates, from Italy, Spain, and Hungary, was carried out [51]. The history of heartworm preventive use was not available for these isolates. Although possible resistance was a concern in these samples, no phenotypic test, such as the MFST, was applied. All eleven isolates were analyzed using the SNP markers previously selected [43] and showed genotypes consistent with susceptibility in isolates from the USA. As in the case of the investigation of the Greek samples, a different resistant isolate, originating from the USA, was used as a control and showed an ML-resistance genotype [51].

The results of the two studies analyzing European strains [44,51] have so far not confirmed ML resistance to heartworm preventives in Europe, but vigilance and testing for resistance would be prudent in cases when dogs become infected, despite apparent use of preventives as recommended.

It is worth mentioning that the area (north-eastern) of Greece where the abovementioned infected dogs were living [44] is characterized by (a) the highest prevalence of canine heartworm in the country, (b) a great problem of stray dogs, (c) large numbers of animals that do not receive adequate veterinary care (and subsequently are not under chemoprophylaxis against heartworm), and (d) a relatively rich wildlife (including canids) population [52,53]. These parameters indicate that a large refugia is present in this specific area and that the establishment of ML resistance in *D. immitis* populations could be considered unlikely. 

The reason that, to date, cases of ML-resistant *D. immitis* infections have not been unequivocally confirmed in Europe is not clear. It is likely that the message of consistent prevention in all dogs, all year round, does not yet have the responsiveness in dog (and cat) owners to the same extent as in the USA (at least in some areas), so selection pressure is probably not as intensive, allowing a large pool of refugia in unprotected dogs. Furthermore, it is also likely that awareness about resistance among veterinarians is not adequately high. This may result in missed diagnoses of resistant infections, considering that, in areas where resistance is not yet well established, such infections (i.e., in dogs under chemoprophylaxis) are likely to be subclinical due to very low numbers of parasites, as only the few resistant parasites would succeed to develop. Thus, it cannot be ruled out that some LOE cases are being missed, especially when the recommended yearly examination for heartworm infection (including both antigen and microfilariae detection) is skipped in dogs under chemoprophylaxis. 

## 10. Is It Likely for Resistance to Expand or Develop De Novo in New Areas? Scenarios for the Future

Given the knowledge that *D. immitis*-resistant strains have occurred and currently circulate in the wider Lower Mississippi area of the USA, it is important to consider the possibility of this phenomenon to geographically expand or develop in new areas of the world. To predict such future scenarios, some decisive factors that determine anthelmintic resistance in general should be taken into consideration [27]. According to the acquired knowledge on the genetic character of ML resistance in *D. immitis*, these factors may be specified in the following points.

i. The genetic polymorphism of the parasites and the genes involved in any existing resistance. There is evidence of significant genetic variability in *D. immitis* populations, a factor that allows the development of drug resistance, depending on the selection pressure [28,54]. It is not known whether multiple genes participate in *D. immitis* resistance to MLs, but the mechanisms of the phenomenon may be complex. Thus far, no specific genes causing resistance have been identified, let alone whether they are dominant or recessive, i.e., show a resistance phenotype in a heterozygote strain, which would be dominant, or only show a resistance phenotype in the homozygous strain, which would be recessive. Furthermore, intermediate forms of expression of resistance are possible, which would be a form of semi-dominance. However, it has been shown that there is a set of SNPs associated with the resistant phenotype and they are predictive [43]. In the field, it is evident that we are dealing with a spectrum of resistant profiles, i.e., some isolates are more or less resistant than others [43,55]. Such a genetic matrix makes resistance development a complicated process. However, it also provides fertile material for the phenomenon to emerge in the case of intense selection pressure. 

ii. The biological traits of the parasite, such as the duration of the lifecycle, the reproduction rate, inbreeding, involvement of intermediate hosts, and lifespan of different life stages. In the case of *D. immitis*, some of these traits favor resistance development while others do not. The relatively long lifecycle (6–9-month prepatent period) opposes the rapid selection, while, in contrast, the high reproductive rate (female heartworms produce millions of microfilariae), the longevity of both adults (5–7 years) and microfilariae (up to 2.5 years) [2], and the relatively small portion of parasites in the short-lived intermediate hosts (that would otherwise represent a significant pool of refugia) facilitate resistance development. Furthermore, there is the likelihood of inbreeding in *D. immitis* because L3 larvae transmitted by a mosquito have a reasonable probability of being siblings or half-siblings, assuming that the mosquito became infected by a blood meal from a single infected dog. Inbreeding will also markedly enhance resistance selection in a parasite such as *D. immitis* [56].

iii. Refugia, i.e., the portion of parasites that escape the selection pressure of drugs. The larger the refugia is, the slower or less probable the resistance development. A low portion of parasites in refugia has been proven to be critically important for the fast development of resistance in other nematode species [27]. In the case of *D. immitis*, the majority of the parasite population of different stages (L3, L4, young adults, adults, and microfilariae) are in the definitive hosts, because of the small number of parasites that infected mosquitoes harbor, and the short lifespan of these insects. Consequently, refugia in the parasite population outside the definitive hosts is minimal. However, refugia that occurs in the definitive hosts is large due to the great number of infected dogs (stray, feral, or dogs with insufficient veterinary care) that are not under any (consistent) heartworm-preventive treatment, and also due to the wild canids that are involved in heartworm epizootiology [2]. This provides grounds for slow resistance development. 

iv. The impact of the resistance genotype on the reproductive fitness and vitality of the parasites and the reversion to susceptibility. It has been shown that, in some cases—for example, in the filarial nematode *O. volvulus*—the presence of alleles that code for resistance seemed to be associated with a loss of reproductive potential [57]. Moreover, there is evidence that when left out of drug pressure, *D. immitis*-resistant strains may partially revert towards their susceptible phenotype [49,58].

v. Prevention strategies other than chemoprophylaxis. In the case of *D. immitis*, as in other vector-transmitted parasites, practices that target vector control and mosquito bite prevention may help the overall reduction of heartworms in a given area, contributing to the reduction of rare resistance alleles [27]. 

vi. Different drugs used for treatment than prevention. Indeed, the advantage of the adulticide treatment (melarsomine dihydrochloride) being different from chemoprophylaxis (MLs) ensures the elimination of adult filariae despite any ML resistance.

All these factors lead to ML resistance emergence, in *D. immitis*, a phenomenon that may be slow to occur in new areas or to expand, without the importation of a resistant population of parasites. In this context, and taking into consideration the current extent of the problem, it is clear that the claim made over sixteen years ago [27], predicting that a significant resistance problem of ML-resistant *D. immitis* is not likely to occur, may still be relevant in Europe. Nevertheless, we now know that this problem is already present, albeit apparently only in a part of the USA, so far. Interestingly, the importation of resistant parasites in a dog to another country (Canada) has been documented [41] and, fortunately, in that case, i.e., a single rescue dog from Louisiana transferred to Ontario, after Hurricane Katrina, the resistance did not appear to spread, perhaps due the dilution of the resistant genotype from this single dog, in the large pool of susceptible parasites and/or the low frequency of transmission occurring in Canada. However, the expansion of resistance by the movement of infected dogs (or mosquitoes) or the de novo emergence cannot be ruled out.

Indeed, local conditions may allow critical selection pressure, e.g., when, in an area, most of the dogs are on preventives, mosquitoes feed on these dogs and a source of unprotected animals (and parasites in refugia) is not available, or very scant. In this case, the main source of infection will be parasites in dogs that have managed to survive and breed because they are resistant [59]. However, this locally occurring resistance is not likely to establish in other areas because the resistant genome would quickly be diluted in the local pool of susceptible parasites if there is a significant number of unprotected hosts, and thus a large refugia. Most likely, this is the reason that, despite the adoption of dogs from the Mississippi Delta to various areas of the USA and Canada, after Hurricane Katrina, of which many were heartworm-positive and probably some with resistant strains [40,41], resistance has not successfully established in those new areas [59] to our knowledge.

However, too much optimism should be avoided. Resistance in *D. immitis* has undoubtedly emerged and this happened in an area with admittedly large refugia [59]. The extent of the problem is not known and, although, to date, it has been unequivocally proven only in the Lower Mississippi region, it cannot be ruled out that cases may have escaped diagnosis or confirmation. Indeed, there is a need for wider surveys in the USA. It should be kept in mind that ML resistance may propagate from an initial geographical point, via animal and vector mobility, to other regions, while it can also emerge as an independent evolutionary process in a new area. As analyzed in the previous sections, resistance is a dynamic and complex phenomenon and prediction of its development cannot be securely achieved. Importantly, as accurately commented by Wolstenholme et al. [59], statistics and the actual extent and distribution of resistance does not matter to the dog owner whose pet animal, despite chemoprophylaxis, became heartworm-positive. For this reason, vigilance and monitoring are essential and, in this context, academics, veterinarians, and owners should work together. 

## 11. What to Expect and How to Monitor

In terms of clinical manifestation, infection by a resistant strain in dogs has a high probability of being asymptomatic [59], especially if resistance is newly emerging or imported in the area and thus resistant strains do not represent the majority of the circulating parasites. The subclinical infection is expected because, in an otherwise protected dog with the consistent administration of preventives, only a few (the resistant) parasites would succeed to develop, and an infection with a very low number of worms usually remains asymptomatic. However, in areas where the phenomenon of resistance is already established, there is good chance that the resistant strains will become more abundant and, in this case, heavy, symptomatic infections in dogs under MLs would be possible. On the other hand, in cats, where heartworm infection usually involves only a few parasites by definition, the low burden of infection by a resistant strain would not alter the classical clinical manifestation of feline dirofilariosis [59]. 

From an epizootiological standpoint, when resistance emerges, it is expected to result in an increase in HW disease incidence in both dogs and cats in an area where prophylaxis coverage is good. This is a plausible scenario as resistance against one of the MLs is likely to manifest as resistance to other MLs. There is evidence that MOX has better performance against resistant strains, but high dosages or long-acting formulations are required for this trait to be expressed [58,60]. 

The first indication for a veterinarian to consider that a case is worth investigating for resistance is when a dog under consistent preventives becomes heartworm-positive. In an integrated and practical description of how to deal with suspected cases, Moorhead et al. [61] proposed an “algorithm” that helps to navigate the veterinarian through the steps needed to solve such dilemmas. This algorithm describes the consecutive actions needed to obtain a clearer picture of the susceptibility nature of the parasites involved in a suspected LOE case. After the confirmation of infection (positive antigen test coupled with *D. immitis* microfilariae presence in the circulation of the dog, or double antigen-positive test [11,62]), the first step towards the investigation of the case is a thorough review of the prevention history of the dog. This includes inquiring about the exact veterinary products used, the intervals between administrations, possible missed or late dosages, prevention year-round or seasonal coverage, the exact doses, and the chance that there was sharing of doses among pets of the same household. This, of course, presupposes that a detailed history and reliable data of the prevention schedule have been recorded and can be accessed. In most cases, this is not feasible and, as proven before, the majority of LOE claims have been ultimately attributed to a lack of compliance [38]. However, there are some cases where such data are available—for example, when the owner is considered absolutely reliable, or when the preventives are administered by the veterinarian (e.g., injectable formulations). If the prevention applied was indeed as recommended and normally would not permit infection, the investigation of resistance can go further only if the dog is microfilaremic, while, in every other case, the veterinarian should simply proceed with the adulticide treatment protocol. In the second step of investigation, the MFST should be applied with a product registered as microfilaricidal, such as Advantage Multi^®^ (Advocate^®^). If the second microfilarial count, 2–4 weeks after microfilaricidal administration, results in a count reduction of >95%, then the parasites can be considered susceptible. If the reduction is <95%, but >75%, then the likelihood of a resistant strain is reduced, but possible. In the opposite case, with <75% reduction, there is indeed a possibility of resistant parasites being involved and there is merit in investigating further such cases in order to monitor the situation and track any expansion or emergence of a resistance problem. It would be important to confirm the resistant nature of the parasites involved in suspected cases both in areas where resistance has been already detected and also in other areas and countries for the sake of the timely and accurate surveillance of the problem. For this purpose, a relatively fast, simple, and inexpensive test that could be performed in the clinic, or at least in routine diagnostic laboratories, would be of great value and companies that are active in the field of veterinary diagnostics should make an effort toward this goal. Until such tests are widely available, samples (microfilariae in blood are adequate) could be obtained and sent to the few institutions and laboratories that are currently in a position to perform the required analyses (genotyping) and identify ML resistance, such as the Institute of Parasitology at McGill University in Canada.

Irrespective of whether there is confirmation of infection by a resistant strain, the treatment protocol should be implicated according to the AHS and European Society of Dirofilariosis and Angiostrongylosis (ESDA) guidelines [11,62], and special emphasis should be given to the following points:

(1). The administration of antibiotics (doxycycline or minocycline) is considered of great value in order to impair the eventual development of the circulating microfilariae to adult worms in a new host, although further confirmation of this effect should be generated [63]. This is the result of removing the filarial endosymbiont *Wolbachia pipientis*, which is critical for the survival, development, and reproduction of *D. immitis* [64]. Furthermore, the elimination of *W. pipientis* results in reduced lung inflammation during the course of adulticide treatment [65,66]. 

(2). The use of MLs licensed as microfilaricidal (Advantage^®^ Multi, Advocate^®^) is recommended for clearing the microfilariae during heartworm treatment, in order to avoid suboptimal effects of MLs, which would promote resistance spreading.

(3). Repellents and long-acting insecticides, such as dinotefuran, permethrin, and pyriproxyfen, can be used in order to avoid mosquito bites and thus disrupt any transmission of the (suspected) resistant strain.

(4). Finally, omitting the pre-adulticidal period, which is 1–3 months according to the proposed heartworm treatment protocol [11], i.e., an option suggested by Bowman and Drake [67] to be effective in eliminating heartworms of all ages in the dog, could be considered by the vet if the general clinical status of the dog and other relevant parameters permit it. This approach would destroy the resistant worms as soon as possible and thus would diminish the chances of the resistance spreading further via mosquitoes [67]. 

Treating a dog infected by ML-resistant heartworms with a “slow kill” protocol [63,66], i.e., by repeated doses of MLs that have been shown to slowly kill adult parasites, is not realistic, as both the microfilariae and adult nematodes would not be susceptible to these drugs. Such an approach would only allow the resistance to be transmitted and perhaps increase in intensity.

## 12. Strategies for Preventing Resistance Development 

For the foreseeable future, chemoprophylaxis of dogs and cats with MLs against dirofilariosis is not negotiable because of the detrimental nature of heartworm disease, its zoonotic potential, and because MLs are the only drug class that is currently available for this purpose. In areas where ML resistance is established and breakthrough infections are confirmed, administration of high-dose formulations of MOX may be of help, as it has been shown that MOX in all forms of products (per os, topical and injectable) has better efficacy against resistant strains [60,68]. Generally, MOX shows higher potency against filarial nematodes, likely in part due to its pharmacokinetic character, which allows better distribution in lipid tissues, lower elimination by the ABC efflux transporters, and a longer half-life [58]. More precisely, for monthly administration, 24 μg/kg (instead of 3 μg/kg) is proposed as the effective oral dose against resistant strains, which is still in the margins of safety for dog breeds that are sensitive to MLs (e.g., Collies) [60]. The long-acting injectable forms of MOX also show better performance against resistant strains [67,69,70], and have the additional advantage of being administered by the veterinarian, removing any concern about owner compliance, and are also effective as microfilaricidal agents against resistant strains [55].

Even though there are no alternative drugs to the MLs currently that would help to combat any ML resistance development, there are, however, measures and strategies that can be implemented in an effort to prevent the development and spread of resistance. In this context, it is important to adopt a tight testing schedule, i.e., at least once every year (preferably every 6 months in areas where LOE cases are reported). This would permit the timely detection of infections despite consistent prevention. The testing procedure is specific and includes both serology and the Knott’s test. It is important to note that the Knott’s test is critical and absolutely necessary in routine annual examinations of dogs under preventatives because even one couple of resistant adults will produce microfilariae but may give a negative antigen test (infection with a very low number of parasites is plausible in the resistance development scenario, as mentioned earlier).

Furthermore, in the course of treating a dog with circulating microfilariae, it is recommended to administer doxycycline to diminish the chance of resistant microfilariae being taken up by mosquitoes and eventually developing to adults in a new host, and thus prevent any spread of ML-resistant parasites. Importantly, the use of mosquito repellents contributes significantly in diminishing the chance of a resistant strain spreading. 

The risk of promoting ML resistance by the application of the so-called “slow kill protocols”, i.e., therapeutic treatment by the use of continuous ML administration (without the adulticidal melarsomine dihydrochloride) to gradually reduce the viability of adult *D. immitis*, has been suggested [16,36]. In this scenario, such a use of MLs may result in suboptimal doses that affect any susceptible worms but have a lesser effect on resistant worms, and thus will promote resistance. Furthermore, in such conditions, microfilariae that are more resistant will be less affected than more susceptible microfilariae, and would have a great transmission advantage [36]. Nevertheless, Wolstenholme et al. [59] suggest that, if a dog is not under prevention and is only infected with susceptible heartworms (which should be evidenced at least by the MFST), the slow kill protocol would serve to promote resistance development only as an extreme and unlikely scenario. In any case, it must be stressed that ML resistance in *D. immitis* can be selected on different stages of the parasites, i.e., the L3/L4 larvae (the target of ML administration as preventives), the microfilariae, and on adult parasites (because of the effects of MLs on their reproductive ability) when MLs are used in the presence of microfilariae and adult parasites.

## 13. Conclusions

Heartworm disease is one of the most severe parasitic diseases in dogs, also affecting other animal species and humans. Prevention is achieved by the administration of MLs, which have been proven to be highly effective and safe when administered according to label instructions. Although most of the cases of infection despite chemoprophylaxis have been proven to be due to a failure to meet heartworm prevention recommendations (e.g., vet/owner compliance, molecule insufficiency due to vomiting, diarrhea, or underdosing), resistant strains of *D. immitis* to MLs have been unequivocally identified.

To date, *D. immitis* resistance to MLs has only been confirmed in dogs infected in the area of the Lower Mississippi, in the USA, and although the extent of the problem is not known, it seems currently not yet a known problem in other countries or regions of the USA. Some suspected cases have been detected in Europe, and despite evidence generated by treatment records, they have not been confirmed by molecular analysis as such.

Resistance development in *D. immitis*, to MLs, is a complicated process, probably involving multiple genes and affected by various factors, such as the potentially large refugia, especially when many pet owners do not put their dogs on preventives, mosquito biting density, and the fact that the drug used to kill adult parasites (adulticide treatment) is not an ML. We do not yet know how quickly resistance can develop in different ecosystems, nor how quickly it can expand once it has developed in a region. However, the phenomenon has already occurred and been verified in multiple studies, so vigilance is warranted. 

Academics, clinical practitioners, and dog owners should be concerned and act together with the goal of monitoring and preventing this phenomenon. This battle starts with proper education and continues with best practices for infection prevention, adequate testing, accurate and prompt diagnosis, accurate investigation of the cases, and the selection of the best treatment protocols. The importance of investigating each suspected resistance case must be stressed, as it will allow the distinction of infections that were established by susceptible parasites due to inadequate prophylaxis from infections caused by truly resistant parasites. This would provide critical information about the actual spread of the phenomenon and its possible expansion or de novo emergence, while, at the same time, it would help to increase practitioners’ and owners’ awareness and compliance [43]. 

Towards the important goal of suspected case investigations, there is a clear need for faster and less expensive methods for the detection of ML resistance. Indeed, further investigations for the development of rapid genetic or in vitro tests that could be performed in the clinic or in laboratories of routine examinations are warranted. Thus, companies involved with diagnostic tests are encouraged to invest their efforts towards this end. At the same time, animal health companies should be encouraged to seek pharmaceutical strategies or vaccines that prevent heartworm disease, without sole reliance on the ML class of anthelmintics for prevention, and this effort should be encouraged by the policies of the registration authorities.

In light of knowledge suggesting that compliance frequently breaks down and that *D. immitis* resistance to MLs has been proven, efforts should be focused on the identification of factors that lead to prevention inconsistency or to veterinarian-administered long-acting treatments, and greater use of mosquito repellents and long-acting insecticides to reduce transmission. Action should be taken to use all possible measures to intercept the occurrence of resistance, targeted towards the better protection of animal and human health.
